# Responses of *Populus trichocarpa* galactinol synthase genes to abiotic stresses

**DOI:** 10.1007/s10265-013-0597-8

**Published:** 2013-11-05

**Authors:** Jie Zhou, Yang Yang, Juan Yu, Like Wang, Xiang Yu, Misato Ohtani, Miyako Kusano, Kazuki Saito, Taku Demura, Qiang Zhuge

**Affiliations:** 1 Key Laboratory of Forest Genetics and Biotechnology, Ministry of Education, Nanjing Forestry University, Nanjing, 210037 China; 2 RIKEN Biomass Engineering Program, Yokohama, Kanagawa 230-0045 Japan; 3 RIKEN Plant Science Center, Yokohama, Kanagawa 230-0045 Japan

**Keywords:** Galactinol synthase, *PtrGolS*, *Populus*, RFO, Abiotic stress

## Abstract

Galactinol synthase (GolS; EC 2.4.1.123) is a member of the glycosyltransferase eight family that catalyzes the first step in the biosynthesis pathway of the raffinose family of oligosaccharides (RFOs). The accumulation of RFOs in response to abiotic stress indicates a role for RFOs in stress adaptation. To obtain information on the roles of RFOs in abiotic stress adaptation in trees, we investigated the expression patterns of nine *Populus trichocarpa* *GolS* (*PtrGolS*) genes with special reference to stress responses. *PtrGolS* genes were differentially expressed in different organs, and the expressions of *PtrGolS4* and *PtrGolS6* were relatively high in all tested organs. The expression levels of all *PtrGolS* genes, except *PtrGolS9*, changed in response to abiotic stress in gene- and stress-type-specific manners. Moreover, short- and long-term stress treatments revealed that induction of *PtrGolS* by salt stress is obvious only in the early period of treatment (within 24 h), whereas water-deficit stress treatments continued to upregulate *PtrGolS* gene expression after two days of treatment, in addition to induction within 24 h of treatment. Consistent with these expression patterns, the galactinol content in leaves increased after four days of drought stress, but not under salt stress. Our findings suggest divergent roles for *PtrGolS* genes in abiotic stress responses in poplars.

**Electronic supplementary material:**

The online version of this article (doi:10.1007/s10265-013-0597-8) contains supplementary material, which is available to authorized users.

## Introduction

Abiotic stresses trigger a series of responses in plants starting with stress perception, leading to activation of signaling pathways and alteration of gene expression levels, resulting in altered plant physiology, growth and development (Loescher 2010). One of the adaptation mechanisms of plant cells is the production of regulatory compounds to protect cells against the effects of osmotic, cold and abiotic stresses. These compounds are known as compatible solutes; mannitol, proline and a large number of soluble oligosaccharides, such as trehalose, raffinose and stachyose (Cuin and Shabala 2008), are examples of compatible solutes responding to abiotic stresses.

RFOs are composed of alpha-galactosyl derivatives of sucrose. The potential role of RFOs in stress tolerance has been intensively studied in seeds, particularly with respect to desiccation tolerance and longevity in the dehydrated state (Bentsink et al. 2000; Bernal-Lugo and Leopold 1992; Downie et al. 2003; Garcia et al. 2006; Peterbauer et al. 2001; Peterbauer et al. 2002; Saravitz et al. 1987). Raffinose is the most common RFO, composed of galactose, fructose and glucose and is synthesized by the donation of galactose from galactinol, a conjugate of *myo*-inositol and galactose, to sucrose by raffinose synthase (RAFS; EC 2.4.1.82). Subsequent additions of galactose units to raffinose result in generation of stachyose, verbascose and other RFOs. Thus, galactinol biosynthesis is likely an important step in the biosynthesis of RFOs. The amount of galactinol was reported to exhibit seasonal changes and to increase under cold conditions (Bachmann et al. 1994, Miao et al. 2007).

Galactinol is synthesized from UDP-D-galactose and *myo*-inositol by galactinol synthase (GolS; EC 2.4.1.123) (Liu et al. 1995; Saravitz et al. 1987). Extensive biochemical studies have characterized GolS in many plant species, including the common bugle (*Ajuga reptans*) (Bachmann et al. 1994), zucchini squash (*Cucurbita pepo*) (Liu et al. 1995; Smith et al. 1991), kidney bean (*Phaseolus vulgaris*) (Liu et al. 1995), soybean (*Glycine max*) (Riberio et al. 2000) and cucumber (*Cucumis sativus*) (Wakiuchi et al. 2003). In addition, molecular biological approaches have accelerated the studies of stress responses of *GolS* genes. Takahashi et al. (1994) reported that *OsGolS* mRNA accumulated in response to cold at 4 °C and to osmotic stress in rice seedlings (*Oryza sativa*). Seven genes belonging to the *GolS* family are present in the genome of *Arabidopsis* *thaliana*. Among them, *AtGolS1*–*AtGolS13* were investigated for their response to abiotic stresses: *AtGolS1* and *AtGolS2* were induced by drought, salt and heat stress and *AtGolS3* was upregulated by cold stress (Taji et al. 2002). *GolS* genes have been identified in other plant species, such as tomato (*Lycopersicon esculentum*) (Downie et al. 2003), *Boea hygrometrica* (Wang et al. 2009), coffee (*Coffea arabica*) (dos Santos et al. 2011), *Salvia miltiorrhiza* (Wang et al. 2012), maize (*Zea mays*) (Zhou et al. 2012), grape (*Vitis vinifera*) (Pillet et al. 2012) and *Medicago falcate* (Zhuo et al. 2012), and most *GolS* genes were reported to be upregulated by abiotic stress treatment. Overexpression of *GolS* genes increases the amounts of galactinol and raffinose with improved abiotic stress tolerance in *GolS*-overexpressing plants (Taji et al. 2002), suggesting that *GolS* genes are good targets for molecular breeding and/or engineering to improve the abiotic stress tolerance of commercial plants.

In this study, we characterized nine poplar *GolS* genes from *Populus trichocarpa* (*PtrGolS1*–*PtrGolS9*). *P. tremuloides* exhibits seasonal alteration in the amount of RFOs: endogenous RFO levels increase in early winter with decreasing temperatures and diminish in spring with increasing temperatures (Cox and Stushnoff 2001). Recently, two GolS isoforms have been isolated from hybrid poplar (*Populus alba* × *grandidentata*), one of which shows seasonal changes in gene expression level (Unda et al. 2012). Thus, RFO levels appear to increase via the regulation of *GolS* expression in cold acclimation of poplars. To further elucidate the roles of RFOs in woody plants, we performed expression analysis of *PtrGolS1*–*PtrGolS9* with special reference to stress response. Our results reveal gene-specific responses of *PtrGolS* genes under different stress conditions, demonstrating diverse roles of *PtrGolS* genes in abiotic stress responses.

## Materials and methods

### Plant materials

*Populus trichocarpa* (Nisqually-1 strain, Tuskan et al. 2006) was used in this study. The young poplar plants were propagated and maintained aseptically on medium containing McCown’s Woody Plant Basal Salt Mixture (pH 5.6; Sigma-Aldrich) under 16-h light/8-h dark conditions at 25 °C. The poplars used for expression analysis and RFO quantification were planted in soil pots (8.5-cm diameter, 14-cm height) and grown in a greenhouse (16-h light/8-h dark, 25 °C) for two months.

### Molecular cloning of *PtrGolS* genes

Sequence information for *PtrGolS* was obtained by performing a BLAST search of the *P. trichocarpa* genome at the Phytozome website (http://www.phytozome.net/) using the AtGolS sequences available in NCBI (http://www.ncbi.nlm.nih.gov/pubmed/). The primers were designed to amplify the coding region of *PtrGolS* genes (Supplementary Table 1) and used for reverse transcription-polymerase chain reaction (RT-PCR) with first strand cDNA template synthesized from total RNA derived from the leaves of poplars. The PCR products were cloned into the pMD 18-T vector (TaKaRa Japan) for sequencing. The experimentally-determined sequences of the *PtrGolS* genes were not identical to the *PtGolS* genes (Supplementary Table 2) described in Unda et al. (2012). The nine genes were submitted to GenBank and the accession numbers are KF496084, KF49608, KF496086, KF496087, KF496088, KF496089, KF496090, KF496091 and KF496092.

### Phylogenetic analysis

The putative amino acid sequences were obtained using the GENESCAN program (http://genes.mit.edu/GENSCAN.html) by submitting the *PtrGolS* cDNA sequences obtained from the molecular cloning analysis. The phylogenetic tree was constructed by the neighbor-joining (NJ) method using the MEGA 5.0 software (Tamura et al. 2011). Bootstrap analysis was performed with 1,000 replicates to evaluate the reliability of different phylogenetic groupings. The obtained tree was drawn using the TreeView software.

### *In silico* prediction of *cis*-acting elements

The 2-kb region upstream of the translation start site of each *PtrGolS* gene was used as the putative promoter region. Prediction of *cis*-acting elements was performed using the PlantCARE (http://bioinformatics.psb.ugent.be/webtools/plantcare/html/) and PLACE (http://www.dna.affrc.go.jp/PLACE/index.html) software to detect well-known abiotic stress-associated elements (ABRE, DRE/CRT and LTRE) (Maruyama et al. 2012; Tuteja 2007).

### Stress and ABA plant treatments

For short-term treatments, young poplars grown on MS agar medium for one month (approximately 9 cm-height) were used. Three plants were transferred to liquid MS medium containing 200 mM NaCl (salt stress treatment), 0.2 % PEG6000 (osmotic stress treatment) or 100 μM ABA. For cold treatment, poplars grown at 25 °C were transferred to a growth chamber adjusted to 4 °C. Whole plants were collected as samples after 0, 2, 10 and 24 h of treatment for salt and osmotic stresses or 24 h of treatment for cold stress and ABA treatment, and immediately frozen in liquid nitrogen. The experiments were repeated three times.

For long-term stress treatment, plants grown in soil pots for two months (approximately 100-cm height) were used. For salt stress treatment, three plants were irrigated with 1 l of 200 mM NaCl solution every day. For drought stress treatment, the water supply was cut off. Leaves were sampled from the treated plants after 0, 2, 4 and 6 days of treatment and immediately frozen in liquid nitrogen after separation into two pieces. One piece was used for measurement of raffinose and galactinol content and the other was used for expression analysis. The experiments were repeated three times.

### Quantitative RT-PCR analysis of *PtrGolS* genes

Total RNAs were isolated from the collected samples using the RNeasy^®^ plant mini kit (Qiagen). To remove contaminating genomic DNA, total RNA was treated with DNase I and then mixed with an equal volume of phenol: chloroform: isoamylalcohol solution, centrifuged at 10,000 rpm, left for 10 min at RT and then the aqueous phase was transferred to a new tube. The RNA was recovered by Dr. GenTLE™ Precipitation Carrier (Takara). For complementary DNA synthesis, 2 μg of total RNA were reverse transcribed using the Transcriptor First Strand cDNA Synthesis Kit (Roche) with oligo (dT) 12–16 primer. Quantitative real-time PCR reactions consisted of 5-μl FS Universal SYBR^®^ Green Master Mix (Roche), 5 pmol forward and reverse primers for *PtrGolS1*–*PtrGolS9* or *EFL4A* genes, 0.5-μl cDNA and water to a total volume of 10 μl. The gene-specific primers were designed using the Primer 5.0 software (Supplementary Table 3). The quantitative PCR analysis was performed with a Lightcycle 480 II instrument (Roche) and the FS Universal SYBR^®^ Green Master Mix (Roche). The reaction conditions were 50 °C for 2 min, 95 °C for 10 min, 45 cycles at 95 °C for 2 min, 62 °C for 30 s and 72 °C for 30 s. The *EFL4A* gene was used as an internal control. The expression ratio was calculated as 2^−ΔΔCq^ (Bustin et al. 2009). The experiment was repeated three times.

### Statistical analysis

The data were analyzed using one-way ANOVA and subsequent post hoc multiple comparison Duncan’s test or Mann–Whitney *U*-test using the SPSS 13.0 software.

### Quantification of raffinose and galactinol content

After stress treatment, leaves were harvested and cut into pieces 5 mm in width, then immediately frozen in liquid nitrogen. Samples were subjected to gas chromatography–mass spectrophotometry (GC–MS) as described by Kusano et al. (2007), with small modifications. Samples were lyophilized using a freeze dryer (Tokyo Rikakikai). After crushing each sample in a Shake Master neo grinder (Biomedical Science) at 1,000 rpm for 5 min, approximately 5 mg (DW) of each sample were weighed and then extracted at a concentration of 2.5 mg (DW) of tissue per milliliter extraction medium (methanol/chloroform/water [3:1:1 v/v/v]) containing 10 stable isotope reference compounds. Five hundred micrograms (DW) were derived, of which 6 μg (DW) were used for GC–MS analysis.

## Results

### *GolS* genes in *Populus trichocarpa*

**Fig. 1:**
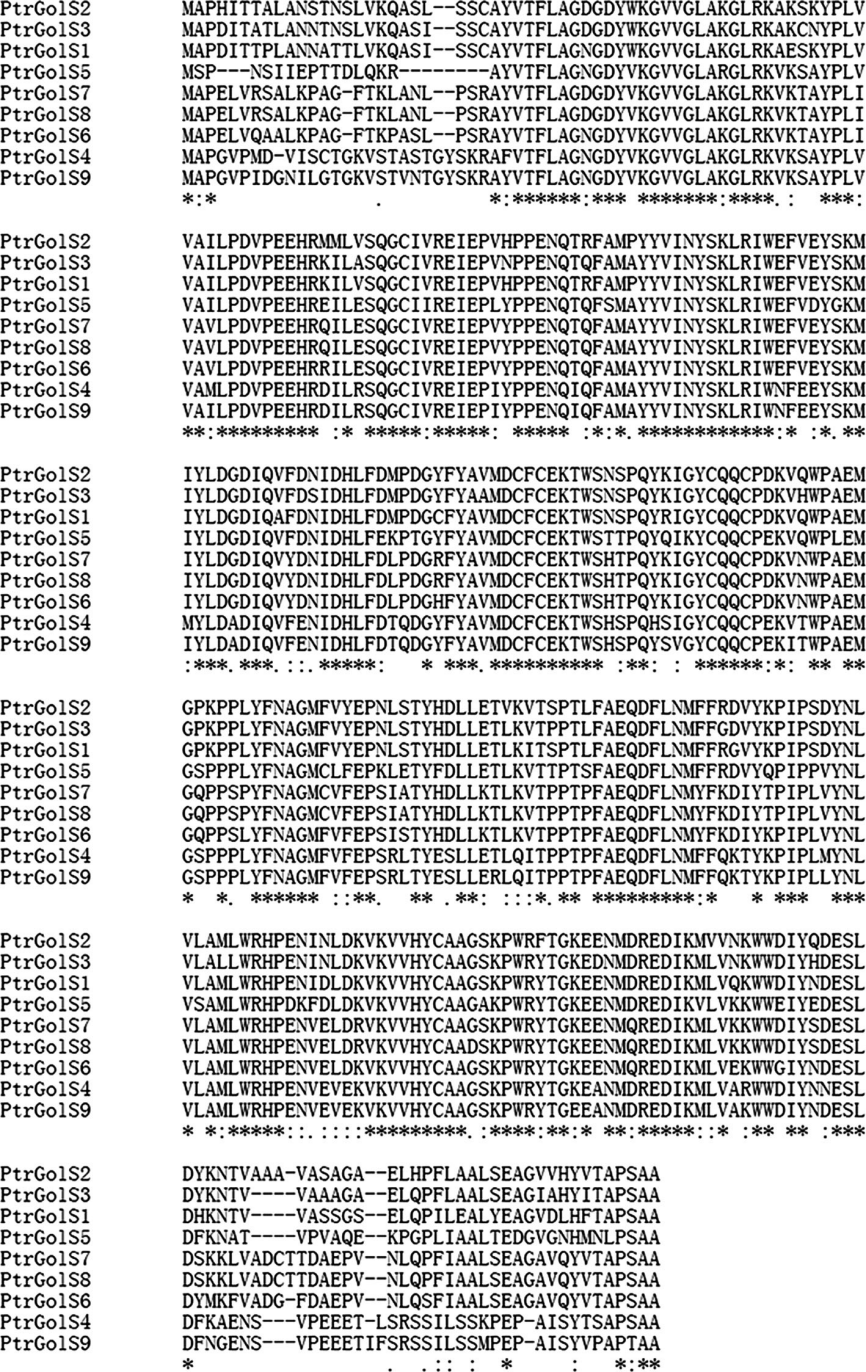
Alignment of deduced amino acid sequences of GolS proteins from *P. trichocarpa* (PtrGolS1–PtrGolS9) generated using the ClustalW software. Conserved residues are indicated by *asterisks* and similar residues by a *colon* or *dot*. The characteristic hydrophobic pentapeptide (APSAA) is located at the end of the sequence

Nine GolS-related genes were identified from the poplar (*P. trichocarpa*) genome database (http://www.phytozome.net/search.php) by a BLAST search using *A. thaliana* GolS. As shown in Table 1, we named the obtained *GolS* sequences *PtrGolS1* to -*9*. The open reading frames of the obtained sequences encode polypeptides of 325–338 amino acid residues. Based on the sequence information, we designed the primer sets to amplify the coding region of each gene and carried out molecular cloning and sequence analysis of the *PtrGolS* genes to experimentally determine the sequences of the genes. All of the predicted GolS protein sequences have the conserved domains of the glycosyl transferase 8 family; only PtrGolS5 shows a single substitution of alanine to leucine in the characteristic hydrophobic pentapeptide (APSAA) at the carboxyl terminal end (Fig. 1).

**Table 1:** Characteristics of *GolS* genes in *Populus* *trichocarpa*

Gene	Locus	Location	Amino acid	Pi	Molecular weight (KDa)
*PtrGolS1*	POPTR_0013s00720	Scaffold_13:354044–355911	334	4.79	38.27
*PtrGolS2*	POPTR_0013s00730	Scaffold_13:362396–364186	337	5.37	38.34
*PtrGolS3*	POPTR_0005s00850	Scaffold_5:390462–392093	334	4.94	38.02
*PtrGolS4*	POPTR_0010s05170	Scaffold_10:6603073–6605381	336	4.98	38.41
*PtrGolS5*	POPTR_0008s10040	Scaffold_8:6253952–6255729	325	4.68	37.51
*PtrGolS6*	POPTR_0010s11210	Scaffold_10:15086593–15088287	325	5.08	38.40
*PtrGolS7*	POPTR_0014s16020	Scaffold_14:8328356–8330354	336	5.09	38.43
*PtrGolS8*	POPTR_0002s19230	Scaffold_2:15155511–15157493	337	5.09	38.43
*PtrGolS9*	POPTR_0008s19370	Scaffold_8:13321787–13323739	338	4.61	38.65

**Fig. 2:**
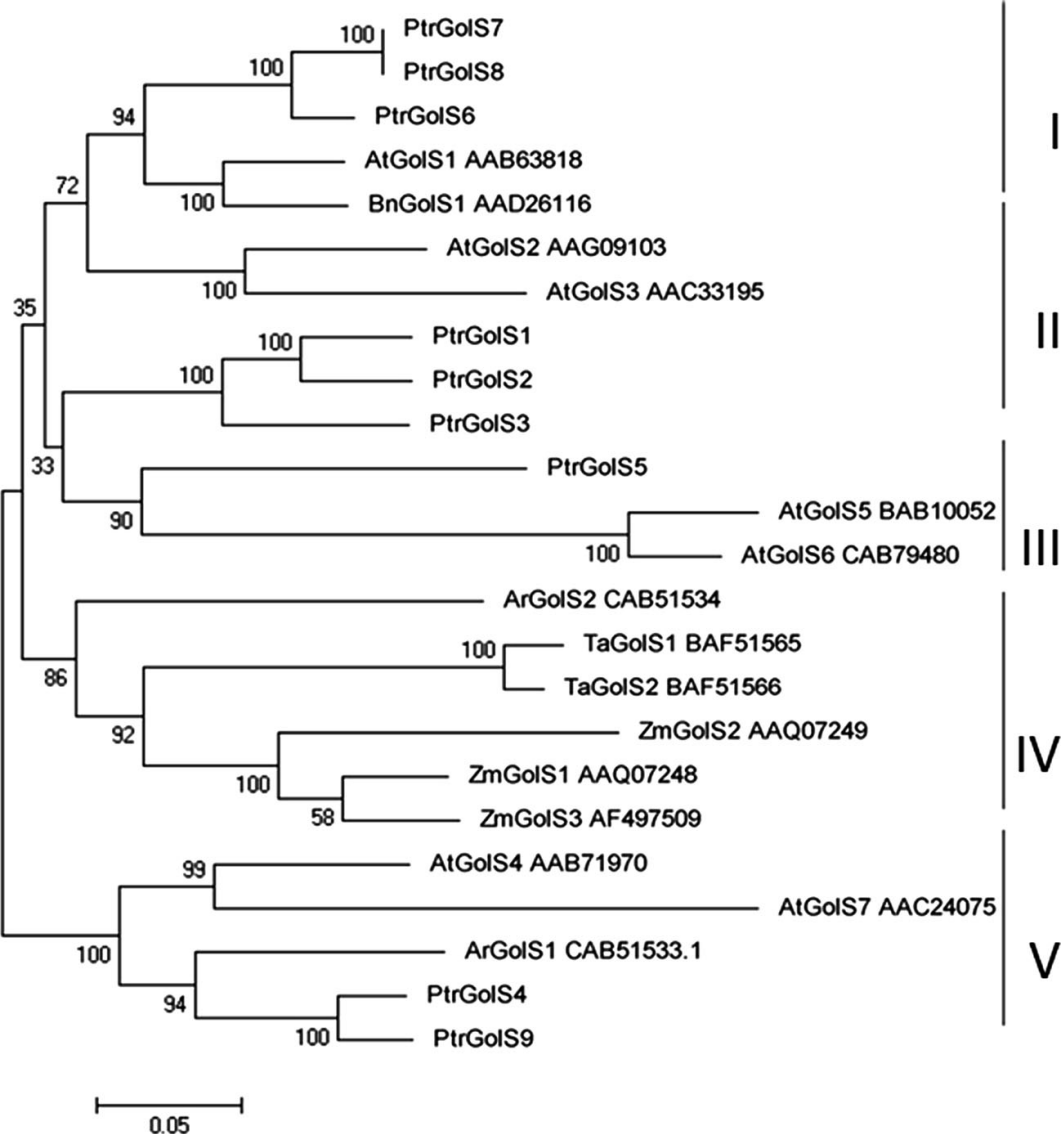
Phylogenetic tree of predicted amino acid sequences of PtrGolS. Unrooted phylogenetic tree of GolS was established by the neighbor-joining method. GenBank accession numbers are provided for GolS proteins other than PtrGolS. Numbers are bootstrap values (1,000 replicates). *Bars* 0.05 amino acid substitutions per site

To elucidate the phylogenic relationship between PtrGolS and known GolS proteins of other plant species, phylogenic analysis was performed using the MEGA 5.0 software. Fifteen full-length amino acid sequences from *A. thaliana* (AtGolS1–AtGolS7), maize (ZmGolS1–ZmGolS3), *A. reptans* (ArGolS1, ArGolS2), *Brassica napus* (BnGolS1) and *Triticum aestivum* (TaGolS1, TaGolS2) were obtained from GenBank. Five clades (I–V) were recognized in the phylogenetic tree (Fig. 2), although the bootstrap values for several clades were low. Branch distribution showed that PtrGolS1–PtrGolS3 (similarity of 89–91 %) grouped with AtGolS2 and AtGolS3 in clade I. PtrGolS6–PtrGolS8 (similarity of 93–98 %) clustered with AtGolS1 and BnGolS1 in clade II. ArGolS2, TaGolS1, TaGolS2, and ZmGolS1–ZmGolS3 grouped together in clade III, which is considered to be a monocot GolS family. ArGolS2 (*A. reptans*) had a low bootstrap value and was not considered part of this clade. PtrGolS5 was in clade IV and PtrGolS4 and PtrGolS9 (similarity of 92 %) were in clade V. PtrGolS proteins were distributed in all clades, except clade III. Thus, the sequences of PtrGolS genes were as divergent as those of other plant GolS-family genes (Philippe et al. 2010).

### Putative *cis*-elements in promoter regions of *PtrGolS* genes

To obtain information on stress-related transcriptional regulation mechanisms of *PtrGolS* genes, an in silico search of *cis*-elements was performed of their putative promoter regions. The PLACE database search showed that abiotic stress responsive elements, such as the ABA responsive element (ABRE) (Zhang et al. 2005), the dehydration and cold responsive elements (DRE/CRT) (Qin et al. 2004) and the low-temperature responsive element (LTRE) (Gao et al. 2002), are present in the promoter region of several *PtrGolS* genes (Table 2). *PtrGolS4* does not possess any abiotic stress responsive *cis*-element, whereas the other *PtrGolS* genes all contain the ABRE element. The DRE/CRT element was found in *PtrGolS1*, *PtrGolS3*, *PtrGolS8* and *PtrGolS9* and LTRE was present in *PtrGolS1* and *PtrGolS7*–*PtrGolS9*. These results suggest that the expression of *PtrGolS* genes is regulated through the corresponding *cis*-elements in response to stress.

**Table 2:** Numbers of *cis*-elements found in promoter regions of *PtrGolS*

Gene	ABRE	DRE/CRT	LTRE
*PtrGolS1*	17	3	6
*PtrGolS2*	18	0	0
*PtrGolS3*	13	5	0
*PtrGolS4*	0	0	0
*PtrGolS5*	2	0	0
*PtrGolS6*	5	0	0
*PtrGolS7*	2	0	2
*PtrGolS8*	6	1	1
*PtrGolS9*	6	1	2

ABRE elements: ACGTG, MACGYGB, TACGTGTC, YACGTGGC, and CCACGTGGA. DRE/CRT elements: RCCGAC, ACCGAC, ACCGAGA, and GTCGAC. LTRE elements: CCGAC, CCGAAA, ACCGACA, and CCGAC. (M = A/C, Y = C/T, R = A/G)

### Expression patterns of *PtrGolS* genes in organs

**Fig. 3:**
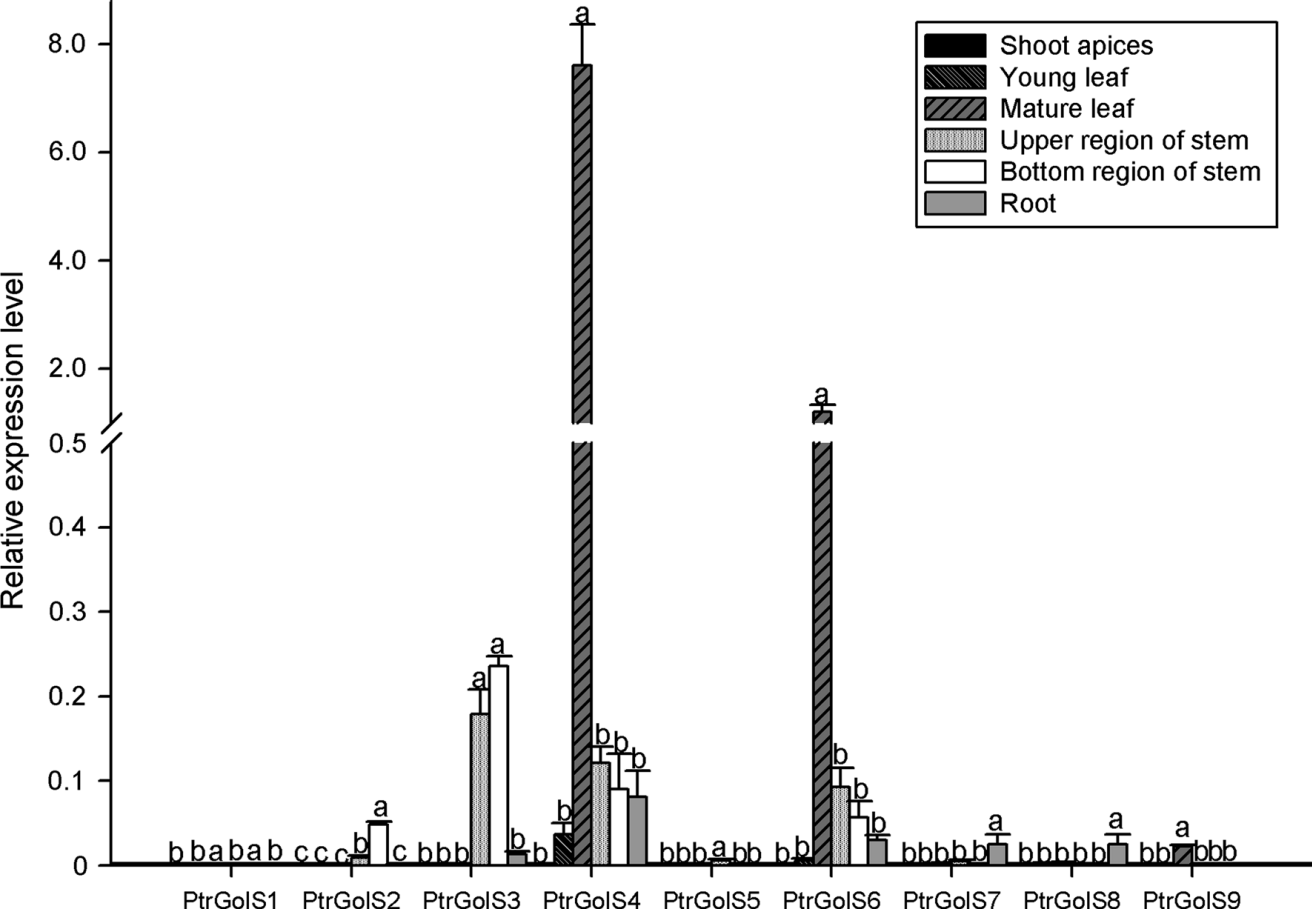
Expression patterns of *PtrGolS* genes in organs by quantitative RT-PCR. Parts of the shoot apices, young and mature leaves, and the upper and lower regions of stems and roots were separately sampled and subjected to total RNA extraction. Results are means ± SE of three replicates. Different *letters* indicate statistically significant differences (post hoc multiple comparison Duncan’s test following ANOVA; *p* < 0.05)

Total RNAs were isolated from shoot apices, young and mature leaves, stems (separated into upper and bottom regions) and roots of the soil-grown poplar plants and subjected to quantitative RT-PCR. *PtrGolS4* and *PtrGolS6* were most abundant in these organs, and *PtrGolS3* and *PtrGolS7* were relatively highly expressed in stems and roots (Fig. 3). *PtrGolS* expression was not abundant in shoot apices. Since the expression levels differed by gene and organ, *PtrGolS* genes might play different roles in the development of poplars.

### Short-term responses of *PtrGolS* expression to abiotic stresses

**Fig. 4:**
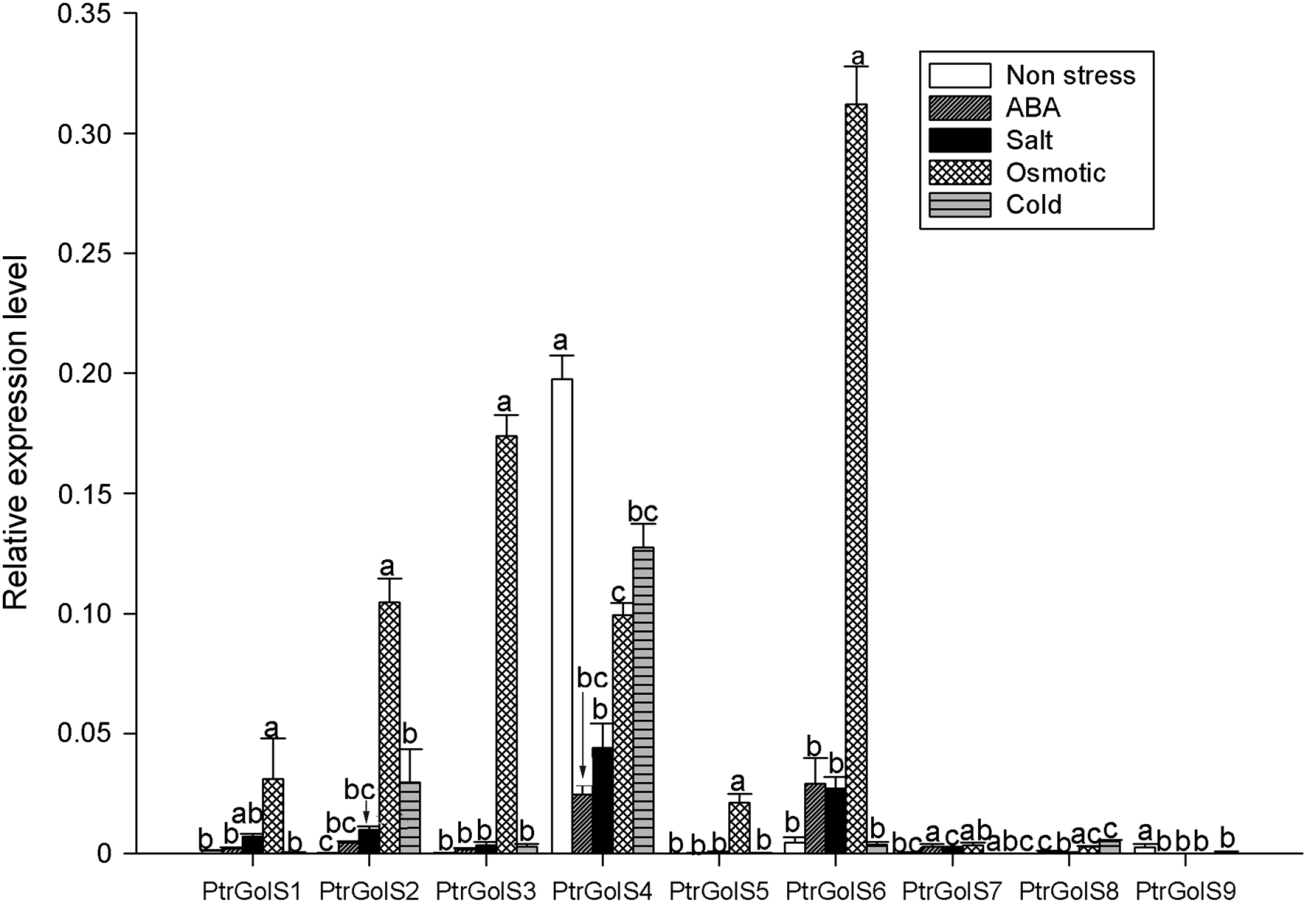
Expression analysis of *PtrGolS* genes in response to salt, osmotic or cold stress and ABA treatment. Poplar plants were cultured in medium containing 200 mM NaCl (salt stress), 0.2 % PEG6000 (osmotic stress) or 100 μM ABA for 24 h. Cold stress treatment was performed by transferring plants to a growth chamber adjusted to 4 °C. Quantitative RT-PCR was performed using total RNA extracted from whole parts of treated plants. Results are means ± SD of three replicates. Different *letters* indicate statistically significant differences (post hoc multiple comparison Duncan’s test following ANOVA; *p* < 0.05)

To examine the stress responses of *PtrGolS* genes, we treated poplar plants with salt, osmotic, and cold stresses, as well as applied ABA for 24 h. As shown in Fig. 4, the responses of *PtrGolS* genes differed according to the stress to which they were exposed. Salt and osmotic stress treatments significantly induced the expression of *PtrGolS1*–*PtrGolS3*, *PtrGolS5* and *PtrGolS6*, whereas cold treatment induced only *PtrGolS2* and *PtrGolS8* (Fig. 4). *PtrGolS2* and *PtrGolS5*–*PtrGolS7* were upregulated by ABA treatment; however, the expression levels were lower than those induced by stress treatment. Notably, *PtrGolS4* and *PtrGolS9* were not induced by any stresses and *PtrGolS4* expression was decreased by the treatments (Fig. 4).

**Fig. 5:**
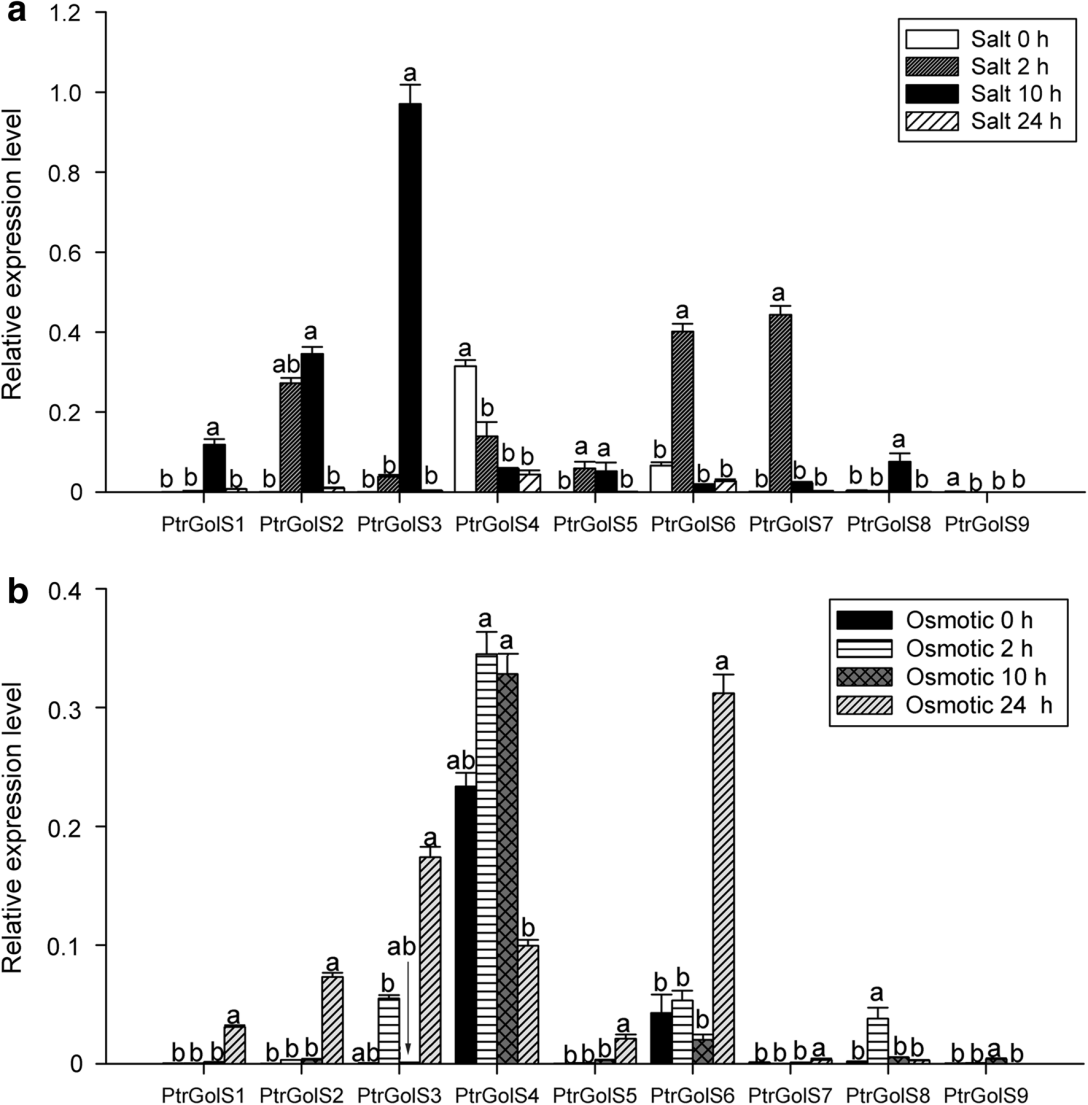
Expression patterns of *PtrGolS* genes under salt or osmotic stress. Poplar plants were cultured in liquid medium containing 200 mM NaCl (**a**) or 0.2 % PEG6000 (**b**) for 2, 10 and 24 h. Quantitative RT-PCR was performed using total RNA extracted from whole parts of treated plants. Results are means ± SE of three replicates. Different *letters* indicate statistically significant differences (post hoc multiple comparison Duncan’s test following ANOVA; *p* < 0.05)

Next we examined the early response of *PtrGolS* expression to salt and osmotic stress (Fig. 5). In the salt stress treatment, *PtrGolS* genes other than *PtrGolS4* and *PtrGolS9* were transiently expressed and peaked at 2 h (*PtrGolS5*–*PtrGolS7*) or at 10 h (*PtrGolS1*–*PtrGolS3* and *PtrGolS8*) (Fig. 5a). The expression levels decreased at 24 h of salt treatment (Fig. 5a). Thus, the early responses of *PtrGolS* genes to salt stress are completed within 24 h. In contrast, the expression patterns of *PtrGolS* in response to osmotic stress differed from those to salt stress (Fig. 5b). *PtrGolS7* and *PtrGolS9* showed no changes in expression level by the osmotic stress treatment. Other *PtrGolS* genes exhibited two types of response: a rapid expression response that resulted in increases in mRNA accumulation at 2 h (*PtrGolS3*, *PtrGolS4*, *PtrGolS6* and *PtrGolS8*), and subsequent upregulation of gene expression at 24 h (*PtrGolS1*–*PtrGolS3*, *PtrGolS5* and *PtrGolS6*). *PtrGolS3* and *PtrGolS6* showed both types of responses (Fig. 5b). Interestingly, *PtrGolS4* mRNA was transiently induced by osmotic stress with a peak at 10 h (Fig. 5b), although *PtrGolS4* expression decreased gradually during salt stress treatment (Fig. 5a). Our data demonstrated that all *PtrGolS* genes, except *PtrGolS9*, change their expression level in response to abiotic stresses, and the expression patterns differ according to stress type.

### Changes in galactinol and raffinose contents and *PtrGolS* expression patterns under long-term stress treatments

**Fig. 6:**
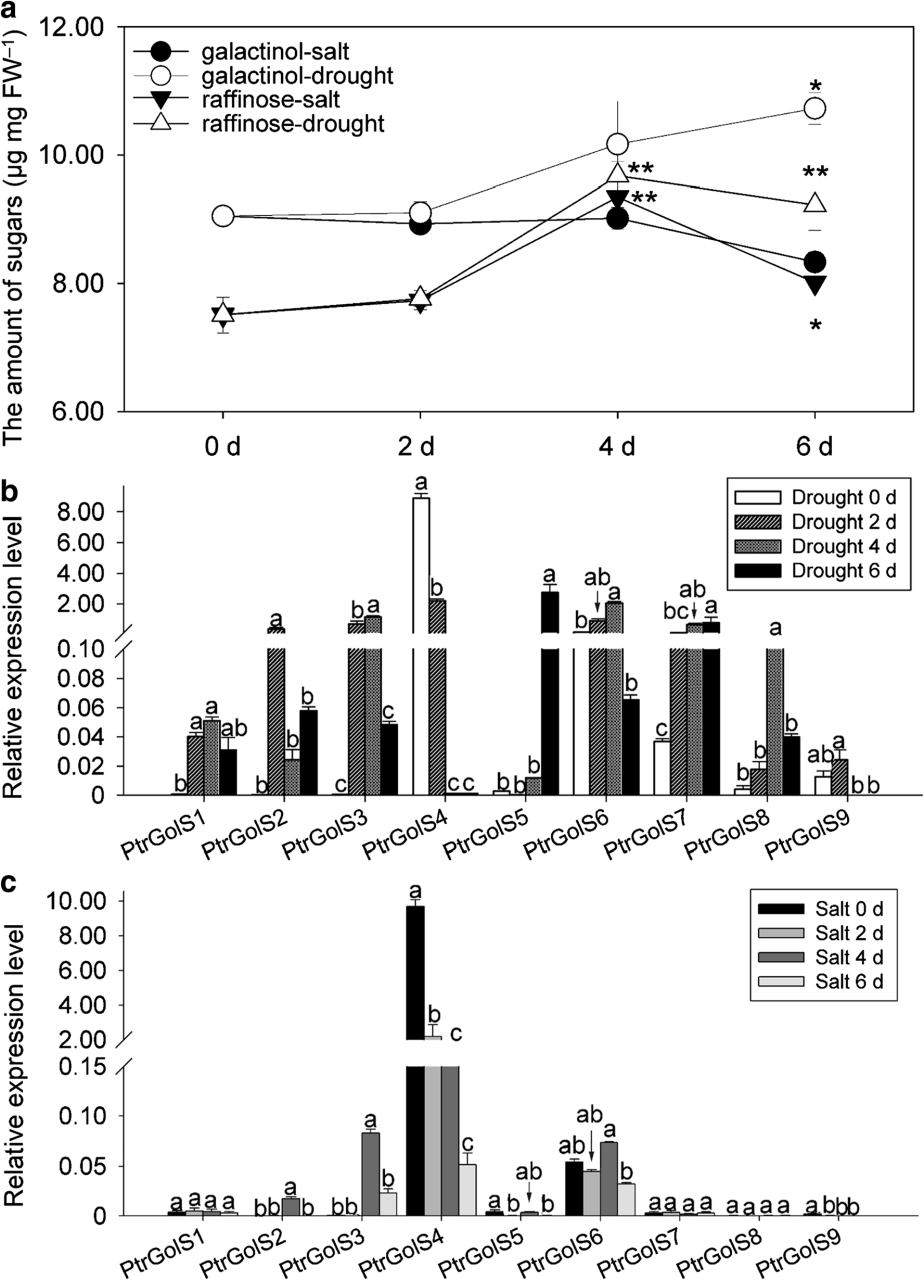
Changes in galactinol and raffinose content and *PtrGolS* gene expression in leaves under long-term salt or drought stress. Plants grown in soil pots for three weeks were subjected to salt or drought stress; leaves were sampled at 0, 2, 4 and 6 days after t he start of treatment. **a** Galactinol and raffinose content determined by GC–MS. *Asterisks* indicate statistically significant increases compared with the content on day 0 (Mann–Whitney *U*-test; *p* < 0.05). (**b** and **c**) Results of quantitative RT-PCR analysis of *PtrGolS* genes under salt (**b**) or drought (**c**) stress. Results are means ± SE of three replicates. Different *letters* in **b** and **c** indicate statistically significant differences (post hoc multiple comparison Duncan’s test following ANOVA; *p* < 0.05)

Finally, we monitored changes in the amounts of galactinol and raffinose and the expression patterns of *PtrGolS* under long-term stress treatment. Plants grown in soil pots were subjected to salt stress by feeding a NaCl solution or drought stress by cutting the water supply, and leaves were sampled after 0, 2, 4 and 6 days. In the salt stress treatment, the leaves turned yellow on the fourth day of treatment while in the drought stress treatment, plants wilted after the fourth day of treatment. Galactinol accumulation clearly increased under drought stress, but not under salt stress, whereas raffinose increased under both stresses (Fig. 6a). The increases in galactinol and raffinose were observed by 4 days of treatment; the raffinose content subsequently decreased (Fig. 6a). mRNA accumulation of *PtrGolS* genes other than *PtrGolS8* and *PtrGolS9* was detected under salt stress, and *PtrGolS2*, *PtrGolS3* and *PtrGolS6* reached a peak on the fourth day (Fig. 6b). *PtrGolS4* was highly expressed but downregulated by salt stress (Fig. 6b). In contrast, *PtrGolS* genes other than *PtrGolS4* were upregulated by drought stress treatment (Fig. 6c). It is suggested that all *PtrGolS* genes, except *PtrGolS4*, may contribute to the accumulation of galactinol and raffinose under drought stress.

## Discussion

Many *GolS* genes have been reported to be induced by abiotic stresses, such as salt, drought and cold (*OsGolS*, Takahashi et al. 1994; *ArGolS1* and *ArGolS2*, Sprenger and Keller 2000; *AtGolS1*–*AtGolS3*, Taji et al. 2002; *MsGolS*, Cunningham et al. 2003; *AmGolS*, Cao et al. 2009), or by biotic stress (*CsGolS1*, Kim et al. 2008; *PtdGolS1, PtdGolS2, PtdGolS6* and *PtGolS3*, Philippe et al. 2010). GolS proteins expressed during seed development are considered to be related to the acquisition of desiccation stress tolerance (Downie et al. 2003). Moreover, GolS is proposed to play distinct physiological roles, synthesizing RFOs for storage and translocating carbon (Sprenger and Keller 2000).

In this paper, we assessed nine putative *GolS* genes from *P. trichocarpa*, *PtrGolS1*–*PtrGolS9* (Table 1). The phylogenetic analysis showed that PtrGolS proteins are distributed in four clades, suggesting that *PtrGolS* genes evolved from four ancestors (Fig. 2; Philippe et al. 2010). Several *PtrGolS* genes were arrayed in pairs (*PtrGolS1* and *PtrGolS2*, *PtrGolS7* and *PtrGolS8, PtrGolS4* and *PtrGolS9*) (Fig. 2), following the idea of multiple gene duplications in the *Populus* lineage (Tuskan et al. 2006). The results of our expression analysis revealed that the *PtrGolS* genes were differentially expressed in an organ-specific manner. The expressions of *PtrGolS4* and *PtrGolS6* were relatively high in all tested organs, while *PtrGolS9* was transcribed only in mature leaves at a low level. Other *PtrGolS* genes were preferentially expressed in stems (*PtrGolS1*–*PtrGolS3*, *PtrGolS5*, *PtrGolS7* and *PtrGolS8*) and/or roots (*PtrGolS3* and *PtrGolS7*) (Fig. 3). Because GolS is involved in RFO synthesis for storage or transport (Sprenger and Keller 2000), *PtrGolS* expression in mature leaves (*PtrGolS4* and *PtrGolS6*) might function in the synthesis of storage RFOs. The *PtrGolS* expression profiles under normal conditions appear to be independent of their distribution in the phylogenetic tree (Figs. 2, 3).

Supporting the possibility that *PtrGolS* is stress-related, multiple stress-related *cis*-elements are detected in the putative promoter regions of *PtrGolS* genes, except for *PtrGolS4* (Table 2). The expression levels of *PtrGolS1*–*PtrGolS8* are significantly influenced by abiotic stress, and each *PtrGolS* responded differently according to different types of stresses (Figs. 4, 5, 6). Almost all *PtrGolS* genes responded to salt, osmotic and drought stresses, whereas cold stress treatment induced only two *PtrGolS* genes, *PtrGolS2* and *PtrGolS8* (Fig. 4). Thus, *PtrGolS2* and *PtrGolS8* might be associated with cold acclimation and seasonal mobilization of carbohydrates, as suggested for GolS from hybrid poplar (Unda et al. 2012). Interestingly, the expression patterns of *PtrGolS4* under stress conditions were distinct from those of the others: *PtrGolS4* mRNA was decreased by 24-h stress treatment and ABA, although the other *PtrGolS* genes were induced (Fig. 4). Further analysis revealed transient upregulation of *PtrGolS4* by osmotic stress during the early treatment period (Fig. 4). These unique patterns for *PtrGolS4* might be related to the fact that no *cis*-elements were found in its promoter region (Table 2). Furthermore, for all *PtrGolS,* the impact of ABA on expression was smaller than that of the stress treatments (Fig. 4), suggesting that the *PtrGolS* genes are also controlled through an ABA-independent pathway during stress responses in poplars.

Regarding long-term stress treatments, galactinol levels were increased only under drought conditions, while raffinose levels were increased under both salt and drought stress conditions (Fig. 6a). The increases in galactinol and raffinose were observed after 4 days of treatment. Consistent with the changes in galactinol content, the expression level of *PtrGolS* was relatively low under salt stress (Fig. 6b), although the induced expression of *PtrGolS* was higher during the early period of salt stress treatment (Fig. 5a). These findings suggest that the expression of *PtrGolS* under salt stress is sufficient to supply galactinol for raffinose biosynthesis, but would not produce excess amounts of galactinol (Fig. 6). Since the expression levels of *PtrGolS3*, *PtrGolS4* and *PtrGolS6* appeared to be relatively high among *PtrGolS* genes, even though *PtrGolS4* was downregulated, they may be major regulators of galactinol synthesis under salt stress conditions. In the case of drought stress, all *PtrGolS* genes other than *PtrGolS4* were relatively highly upregulated, as compared to salt stress (Fig. 6b, c). Thus, under drought stress, the upregulation of *PtrGolS* would result in a significant increase in the amount of galactinol.

In conclusion, we propose different roles for *PtrGolS* genes in stress responses in poplars: *PtrGolS2* and *PtrGolS8* are involved in cold acclimation (Fig. 4) and *PtrGolS3, PtrGolS4* and *PtrGolS6* mainly contribute to galactinol production under long-term salt-deficit conditions (Fig. 6), whereas all *PtrGolS* genes would function in galactinol production under long-term water-deficit conditions (Fig. 6). Rapid responses to salt and osmotic stresses were also detected for other *PtrGolS* genes (Fig. 5), which suggests the involvement of other *PtrGolS* genes in salt- and water-deficit responses, particularly early responses. Many studies have reported that GolS isozymes differ in their enzymatic properties as well as physiological conditions for their activities (Bachmann et al. 1994; Liu et al. 1995; Riberio et al. 2000; Unda et al. 2012). Further characterization of PtrGolS proteins is thus required to elucidate the roles of GolS in RFO biosynthesis in response to abiotic stresses.

## Electronic supplementary material

Below is the link to the electronic supplementary material.

Supplementary material 1 (DOC 57.5 kb)

## Abbreviations

ABA: Abscisic acid
ABRE: ABA responsive element
DRE/CRT: Dehydration and cold responsive element
GolS: Galactinol synthase
LTRE: Low-temperature responsive element
MS: Murashige and Skoog
NJ: Neighbor-joining
RT-PCR: Reverse transcription-polymerase chain reaction
RFOs: Raffinose family of oligosaccharides
