# Supplementary material for: Responses of Populus trichocarpa galactinol synthase genes to abiotic stresses
Source: J Plant Res. 2013 Nov 5;127(2):347–58. doi: 10.1007/s10265-013-0597-8 (PMC3932401; doi:10.1007/s10265-013-0597-8)
Supplement: Supplementary file 1 — Supplementary material 1 (DOC 57.5 kb) [file 10265_2013_597_MOESM1_ESM.doc]

electronic supplementary material

**Table 1.** The primers for coding region of *PtrGolS* genes

| **Gene** | **Primer sequence** |
| --- | --- |
| *PtrGolS1* | F: 5'-ATGGCTCCTGATATCACTACTCCTC-3' |
|  | R: 5'-TTAAGCGGCAGATGGGGCGGTAATG-3' |
| *PtrGolS2* | F: 5'-ATGGCTCCTCATATTACAACTGCCC-3' |
|  | R: 5'-CTAAGCGGCGGATGGGGCGGTAACA-3' |
| *PtrGolS3* | F: 5'-ATGGCTCCTGATATTACTGCTACTC-3' |
|  | R: 5'-TTAAGCGGCAGATGGGGCGGTAATG-3' |
| *PtrGolS4* | F: 5'-ATGGCTCCAGGAGTGCCCATGGATG-3' |
|  | R: 5'-TTAAGCAGCAGATGGCGCAGAGGTA-3' |
| *PtrGolS5* | F: 5'-ATGTCTCCCAATTCCATAATCGAGC-3' |
|  | R: 5'-TTAAGCTGCAGATGGTAGATTCATG-3' |
| *PtrGolS6* | F: 5'-ATGGCTCCTGAGCTTGTCCAGGCTG-3' |
|  | R:5'-TGCAGTGACGGGGTAGGCGACGAATC-3' |
| *PtrGolS7* | F: 5'-ATGTCACCAAATGCCATTATCGAGC-3' |
|  | R: 5'-TTATGCTGCCTATGGTAGATTTCTA-3' |
| *PtrGolS8* | F: 5'-ATGGCTCCTGAGCTTGTACGGTC-3' |
|  | R: 5'-CTAGGCAGCAGATGGGGCAGTGA-3' |
| *PtrGolS9* | F: 5'-ATGGCCCCAGGAGTGCCTATAGATG-3' |
|  | R: 5'-TTAAGCAGCAGTTGGTGCAGGGACG-3' |

**Table 2**. *PtrGolS* genes in this study

| **Gene name**  **in this study** | **Gene name in**  **Unda et al (2012)** | **Gene ID in JGI*** | **Location of gene in the genome** |
| --- | --- | --- | --- |
| *PtrGolS1* | *PtGolS3* | POPTR_0013s00720 | scaffold_13: 354044 - 355911 |
| *PtrGolS2* | *PtGolS4* | POPTR_0013s00730 | scaffold_13: 362396 - 364186 |
| *PtrGolS3* | *PtGolS5* | POPTR_0005s00850 | scaffold_5: 390462 - 392093 |
| *PtrGolS4* | *PtGolS2* | POPTR_0010s05170 | scaffold_10:6603073 - 6605381 |
| *PtrGolS5* | *PtGolS8* | POPTR_0008s10040 | scaffold_8: 6253952 - 6255729 |
| *PtrGolS6* | *PtGolS7* | POPTR_0010s11210 | scaffold_10:15086593-15088287 |
| *PtrGolS7* | *PtGolS9* | POPTR_0014s16020 | scaffold_14: 8328356 - 8330354 |
| *PtrGolS8* | *PtGolS6* | POPTR_0002s19230 | scaffold_2:15155511-15157493 |
| *PtrGolS9* | *PtGolS1* | POPTR_0008s19370 | scaffold_8: 13321787-13323739 |

* DOE Joint Genome Institute, http://www.phytozome.net/poplar.php

**Table 3.** The gene-specific primers of *PtrGolS* genes for quantitative RT-PCR analysis

| **Gene** | **Primer sequence** |
| --- | --- |
| *PtrGolS1* | F:5'-TGTTGATCTCCACTTCACCG-3' |
|  | R:5'-CAGTAGGCTTTTCAGGAACC-3' |
| *PtrGolS2* | F:5'-ACAGGGATGGGATATTGCTC-3' |
|  | R:5'-GTTAGTCAGAGCAAATCCCA-3' |
| *PtrGolS3* | F:5'-GGTGATTGTGGAGCTAGTAG-3' |
|  | R:5'CCAGACTTGGAAGAAAACTC-3' |
| *PtrGolS4* | F:5'-AAACTGGTGTAATCCCATCC-3' |
|  | R:5'-CACCTTAAATAACACCACAG-3' |
| *PtrGolS5* | F:5'-CTAGCTAGCTAGTGTGTTTGGAGC-3' |
|  | R:5'-CCACTTTCTTCAGTGATCACAATCAAACG-3' |
| *PtrGolS6* | F:5'-GAGAGAGAGCAGAGCAGAGC-3' |
|  | R:5'-CACCATTGCTCCAGTAGCTT-3' |
| *PtrGolS7* | F:5'-ATACCTCTAGTGTGCTCGGG-3' |
|  | R:5'-CACAACTCATGACATGCTAATAACGTGC-3' |
| *PtrGolS8* | F:5'-GGAAGAAAGAAGGATCAAAG-3' |
|  | R:5'-AAACAAGAAAAGAGGGAGTG-3' |
| *PtrGolS9* | F:5'-CTTCTTAGTTAAGAGAGTTTAAAATC-3' |
|  | R:5'-TAGTATAAAAGTTCAAATACTGTAATAC-3' |
